# Psychiatric admissions from crisis resolution teams in Norway: a prospective multicentre study

**DOI:** 10.1186/1471-244X-13-117

**Published:** 2013-04-18

**Authors:** Nina Hasselberg, Rolf W Gråwe, Sonia Johnson, Jūratė Šaltytė-Benth, Torleif Ruud

**Affiliations:** 1 Department of Research and Development, Division Mental Health Services, Akershus University Hospital, Lørenskog, Norway; 2 Institute of Clinical Medicine, University of Oslo, Oslo, Norway; 3 Department of Research and Development, Alcohol and Drug Treatment Health Trust in Central Norway, Trondheim, Norway; 4 Norwegian Centre for Addiction Research, Institute of Clinical Medicine, University of Oslo, Oslo, Norway; 5 Department of Mental Health Sciences, University College London, London, UK; 6 HØKH, Research Centre, Akershus University Hospital, Lørenskog, Norway

**Keywords:** Acute psychiatric services, Crisis resolution teams, Mental health services, Admission

## Abstract

**Background:**

Crisis resolution teams (CRTs) provide intensive alternative care to hospital admission for patients with mental health crises. The aims of this study were to describe the proportions and characteristics of patients admitted to in-patient wards from CRTs, to identify any differences in admission practices between CRTs, and to identify predictors of admissions from CRTs.

**Methods:**

A naturalistic prospective multicentre design was used to study 680 consecutive patients under the care of eight CRTs in Norway over a 3-month period in 2005/2006. Socio-demographic and clinical data were collected on the patients, and on the organization and operation of the CRTs. Logistic regression analysis for hierarchical data was used to test potential predictors of admission at team and patient level.

**Results:**

One hundred and forty-six patients (21.5%) were admitted to in-patient wards. There were significant differences in admission rates between the CRTs. The likelihood of being admitted to an in-patient ward was significantly lower for patients treated by CRTs that operated during extended opening hours than CRTs that operated during office hours only. Those most likely to be admitted were patients with psychotic symptoms, suicidal risk, and a prior history of admissions.

**Conclusions:**

Extended opening hours may help CRTs to prevent more admissions for patients with moderately severe and relapsing mental illnesses. Patients with severe psychosis seem to be difficult to treat in the community by Norwegian CRTs even with extended opening hours.

## Background

Crisis resolution teams (CRTs) are specialized mobile teams intended to provide a rapidly available and intensive short-term home treatment to prevent admission to in-patient wards for patients experiencing an acute mental health crisis. Their target group is patients who would otherwise be admitted to in-patient wards [1,2].

In 2000, the UK government established CRTs nationally [3], and in 2005, the national health authorities of Norway decided to implement the CRT model at all community mental health centres (CMHCs) [4]. Recent studies, mostly from the UK, indicate that the introduction of CRTs may be associated with a reduction in hospital admissions [5-11], although the evidence is not wholly consistent. Tyrer et al. found that the introduction of CRTs was associated with an increase in involuntary admissions and a decrease in voluntary admissions [12]. Jacobs and Barrenho found no evidence that the CRT policy *per se* made any difference to admissions, taking into account other possible explanatory factors such as changes in primary care trusts (PCTs) before and after the introduction of CRTs, and cross-sectional changes in PCTs with and without CRTs [13].

Although CRTs are seen as an alternative to in-patient admission, some CRT patients are, nevertheless, admitted to in-patient wards. Studies over the last decade have found that about one-fifth of CRT patients diagnosed with acute mental health problems were admitted to in-patient wards [9,14]. To our knowledge, only one study in the last decade has presented data about factors associated with hospital admission from CRTs [15]. This study found that the patients most likely to be admitted were those who were uncooperative at the initial assessment, at risk of self-neglect, with a history of involuntary admissions, and who were assessed outside office hours or in hospital casualty departments. It also found that the particular CRT delivering the service was a consistent determinant of hospital admission [15], although this variable was not otherwise specified in their study. Older studies of mobile home-treatment services before 2000 identified severe mental illness, previous hospital admission, suicide risk, and referral route (referral by the police, legal system, or health professionals) as predictors of in-patient admission [14,16,17].

Studies on CRTs are sparse and most are from the UK. There is a need for studies from other countries. A better understanding of the variables associated with admission from CRTs in routine practice may help with further service planning and development of both CRTs and psychiatric in-patient wards.

The aims of our study were: (a) to describe the characteristics of Norwegian CRTs and to identify if there were differences in admission practices between them; (b) to describe the proportions and characteristics of patients admitted to in-patient wards from CRTs; and (c) to identify team and patient predictors of admissions from CRTs.

## Methods

### Setting

Norway has a total population of five million people. Compared to the UK and many other countries, Norway has more rural areas and a lower population density. The standard of living is generally high. Mental health service provisions for adults consist of primary care and specialized mental health services. The primary health-care services run by the 429 municipalities consist of general practitioners (GPs) and primary care mental health teams, usually staffed by psychiatric nurses, social workers, and occupational therapists. Many municipalities have residential care, day-care centres for people with mental health problems, and ambulatory care. The specialized mental health services run by the 20 health authorities and trusts include 75 CMHCs, hospitals with acute psychiatric wards and specialized wards, and psychiatrists/psychologists in private practice. The CMHCs usually consist of outpatient clinics, in-patient wards, day care, and one or more specialized teams (case management teams, early intervention teams for first-episode psychoses, CRTs, and assertive community treatment teams). Specialized services for substance abuse are usually organized as part of the specialized mental health services run by the health authorities.

In 2005, the Norwegian national health authorities decided to implement CRTs in all CMHCs. The aim was to increase the availability and mobility of specialized mental health services to manage episodes of acute mental health crisis outside in-patient wards [4].

In Norway, patients may be admitted either to in-patient mental health care in acute psychiatric wards in hospitals or to in-patient wards in CMHCs (both referred to below as in-patient wards). CRTs can admit patients to both. In 2008, there were 61 CMHCs beds and 21 acute psychiatric ward beds in hospitals per 100,000 inhabitants [18]. Only acute psychiatric wards in hospitals have a mandatory duty to accept emergency admissions. Most of the in-patient CMHC wards are not certified for involuntary treatment.

Unlike the UK system, where CRTs gate-keep the beds, the Norwegian CRTs do not have a gate-keeping role. In-patient referrals are made by GPs, casualty clinics, CRTs, or other CMHC teams or clinical units.

### Sample

The sample consisted of 680 consecutive patients aged 18 years or older who presented with a mental health crisis in a face-to-face consultation in one of eight CRTs during a 3-month inclusion period in 2005/2006. Detailed descriptions of patients, CRT teams, and the content of treatment have been reported previously [19,20].

The eight participating CRTs comprised all of the CRTs in Norway at that time, except one that had recently carried out a study of its own [21]. The populations of the eight catchment areas ranged from 65,000 to 115,000. There were CRTs from each of the five health regions of the country, from both urban and rural areas. Norway is a homogeneous society with relatively minor differences in living standards between urban and rural areas. None of the catchment areas can be characterized as highly deprived. None of the CRTs operated with a rapid response, 24/7 availability, nor had gate-keeping functions for acute wards [19]. Compared to the UK, the Norwegian CRTs provide less intensive and less out-of-office care (7.4% of the patients had had consultations more than twice weekly) [20].

Referral practices to in-patient wards from the CRTs varied between the teams. Some CRTs wanted GPs and casualty clinics to admit patients directly to the in-patient wards when there was no doubt that the patient should be admitted. Others wanted involuntary admissions to go directly to hospitals during the CRT’s opening hours.

By 2010, CRTs had been established at 51 of the 75 CMHCs in Norway. Thirty of these operated only during office hours, and only one had 24-hour availability [22]. Half of the teams established in 2008 had no full-time psychiatrist [18]. This indicates that the CRTs may not have changed significantly since our data were collected, and that our data are still representative.

### Data collection

A registration form was used to record information about the patients and their treatment from admission to discharge. The form was developed and piloted in collaboration with the clinical staff of the CRTs from 2003 to 2005. The clinicians in each team jointly completed the form for every patient who received at least one consultation. The inclusion period started at different time points for different CRTs (between November 2005 and April 2006). The CRTs included all patients referred during the 3-month inclusion period, and where necessary the period was extended to obtain a minimum of 60 patients to provide a reasonable sample of patients from each team for a comparative data analysis. Each team leader also completed a questionnaire about the organization and operation of the team.

### Measures

Data was obtained on socio-demographic and clinical characteristics, contact during the 48 hours prior to referral to the CRT, and the referral process. Type and severity of psychiatric problems and level of functioning were assessed using the International Statistical Classification of Diseases and Related Health Problems, 10th revision (ICD-10) for diagnoses [23], the Health of the Nation Outcome Scale (HoNOS) [24], and Global Assessment of Functioning Scale (GAF) [25].

The HoNOS consists of 12 problem area subscales, each of which rates problems using the options of 0 (no problem), 1 (minor problem requiring no action), 2 (mild problem, but definitely present), 3 (moderately severe problem), to 4 (severe to very severe problem). The subscales relevant to, and included in, this study were non-accidental self-injury (HoNOS 2), problems with drinking or drug-taking (HoNOS 3), problems with hallucinations and delusions (HoNOS 6), and problems with depressed mood (HoNOS 7). Clinicians received 4 hours’ training in the use of the HoNOS, based on the training model developed in the UK. An earlier study, which used the same training for clinicians, showed acceptable inter-rater reliability (intra-class correlation coefficient of 0.60–0.89) for the HoNOS subscales used in this paper [26]. Several studies have indicated moderately high internal consistency and low item redundancy for the HoNOS sum score, and therefore support the instrument as an adequate measure of symptom severity [27].

We used a split version of the GAF consisting of two scales ranging from 1 to 100 for symptom severity and functional impairment [25]. Clinicians were familiar with the use of the GAF because it is a routine measure in Norwegian mental health services. Jones et al. found the GAF scales’ reliability and validity to be satisfactory [28]. Söderberg et al. suggested that when staff use patients’ GAF scores to measure changes and outcomes, it might be necessary to use several raters for each individual patient [29]. In our study, two or more raters usually collaborated to complete the registration form, including the GAF score.

Suicide risk was coded as: (i) no suicidal thoughts or plans; (ii) passive death wishes or suicidal thoughts without concrete plans; (iii) concrete suicidal plans or self-injury, but no death intention; and (iv) self-injury with death intention. This scale was designed in collaboration with the National Centre for Suicide Research and Prevention [30].

Some patients were brought to the attention of the CRTs by reports from seriously concerned family, friends, or neighbours, from the staff at casualty departments, CMHCs or primary care mental health teams, or from GPs. The police were asked for assistance in a crisis if there was a risk of harm to the patient or others, or if the crisis was occurring in the community and the public was concerned for their safety. Some of these patients were uncooperative during the initial assessment.

The questionnaire to the team leaders sought information on opening hours, whether there was a full-time doctor on the team, number of patients treated during the inclusion period, number of team members, focus on home treatment, whether there was a psychosis team in the catchment area, whether the CRT had the authority to admit patients to acute in-patient wards, and whether they accepted self-referrals.

### Approval from authorities and contributions from user groups

The study was approved by the Regional Committee on Ethics in Medical Research, the Norwegian Data Inspectorate and the Directorate of Health. Written consent was not requested because the Regional Committee on Ethics in Medical Research agreed that, for ethical reasons, it was important to include all patients in need of acute treatment, especially those with severe mental illnesses who probably would not have had the capacity to give written consent.

The in-patient wards admit patients from areas with and without CRTs, and the Norwegian Data Inspectorate did not allow us to record in which municipalities the patients lived. We were therefore unable to analyse differences in admission rates between wards with and without CRTs in their areas.

Representatives of the user organizations, Mental Health Norway and The National Association for Relatives in Mental Health, participated in a reference group and in the workshops for the planning and preparation of the study.

### Data analysis

HoNOS scales with missing values were set to 0. This was considered the most probable rating, based on the skewed distribution, where most patients rated 0, and the assumption that clinicians most easily forgot to mark the rating when there was no indication of problems. It was also chosen over imputation because it was the most conservative way to measure the severity of patients’ mental health problems. Diagnoses were missing for 53.5% and 17.4% of the patients in two of the teams and for 3.4–10.4% in the other six teams. In Norway, only physicians, psychiatrists, and psychologists are authorized to make ICD–10 diagnoses. The teams with the highest number of missing values on the diagnosis variable operated without a physician, psychiatrist, or psychologist as a regular member of the team. In these teams, diagnoses were made by physicians who were not part of the team. For this reason, the HoNOS scales were used instead of diagnoses in the analysis of the type and severity of the psychiatric problems.

Socio-demographic and clinical characteristics were described as frequencies and percentages or means and standard deviations, as appropriate. Differences between those who were and were not admitted to in-patient wards were analysed by chi-squared tests for categorical variables, and independent samples *t*-tests for continuous variables, including skewed variables, as comparison with a non-parametric alternative exhibited only marginal differences in *p*-values.

To explore the potential predictors for admission vs. non-admission, data on both patient and team levels were used. The selection of potential predictors on both levels was based primarily on earlier studies referred to in the background section [14-17]. The significant covariates in the bivariate analyses between patients admitted and not admitted were used as potential predictors, while age and gender were used regardless of their significance. Attempted suicide, deliberate self-harm and GAF were not included in the regression analysis for hierarchical data even though they were significant. This was because of the risk of interaction effects between these and other similar variables, such as the HoNOS subscales.

To assess the association between admission status and potential predictors on both levels, a hierarchical logistic regression model with random effects for intercepts was fitted (SAS GLIMMIX procedure). Such a model takes possible correlations between members of the same cluster (i.e., team) into account, and might protect against false significant findings. Crude odds ratios were calculated by bivariate analyses, and then adjusted by estimating a multivariate model. The model with all selected predictors included was reduced by a stepwise selection procedure with entry and stay probabilities close to one [31]. This method produces a sequence of models starting with the null model (no predictors) and ending with the full model (all potential predictors included). At each step, Akaike’s information criterion (AIC) was calculated and the model with the lowest AIC value chosen as the final one. Patient-level interactions between the following variables were considered: delivered to CRT by the police and physical attacks on others, suicide risk and depressive symptoms, and psychiatric admission in the past 12 months, and psychotic symptoms. One team-level interaction was considered: number of team members and number of patients treated. No cross-level interactions were included. The statistical analyses were conducted using software SAS version 9.2 for Windows (SAS Institute Inc., Cary, NC) and SPSS software version 18.0 for Windows (SPSS Inc., Chicago, IL). The significance level was set at 5%. Holm’s method [32] was used in the regression analysis to control the family-wise error rate. The original *p*-values were presented in a table, but the predictors that remained significant after the adjustment were singled out.

## Results

There were three related research questions in this study: to describe the characteristics of Norwegian CRTs and identify whether there were differences in admission practices between them, to describe the proportions and characteristics of patients admitted to in-patient wards from CRTs, and to identify team and patient predictors of admissions from such teams.

### Characteristics of the CRTs and differences between teams in admission practices

Table 1 presents some characteristics of the organization and operation of the eight CRTs in this study. The CRTs were not available 24 hours a day, 7 days a week, but four of the CRTs had extended their opening hours to the evenings and weekends. The number of team members varied substantially (range = 4–19 full-time equivalent staff members or 0.5–2.0 staff members per 10,000 inhabitants). Three teams did not have a full-time doctor as a part of the team, all but one team accepted self-referrals, and half of the teams had the authority to admit patients to acute in-patient wards (the rest had to refer patients to such wards). None of the CRTs had gate-keeping functions for the acute beds in their catchment area.

**Table 1:** Characteristics of CRTs (n = 8)

**Team-level variables**	
Opening hours: n	
24/7 (24 hours/day, 7 days/week)	None
Team operates office hours (37.5 hours/week)	4
Team operates extended hours (70 hours/week)	1
Team operates extended hours (75 hours/week)	2
Team operates extended hours (86 hours/week)	1
Number of team members (FTE) mean (range)	9.1 (4.3–19.2)
Full-time doctor as a part of the team: n	5
Number of patients included: n (median)	46–147 (80)
Focus on home treatment: n	5
Psychosis team in the catchment area: n	5
Accept self-referral: n	7
Authority to admit patients to acute in-patient wards: n	4

There were significant differences between the CRTs in the overall in-patient ward admission rate (range = 13.3–37.3%). The CRTs with high admission rates had patients with more severe mental health problems on average (GAF symptom and functioning, *p* < 0.001, HoNOS total moderately to very severe problems, *p <* 0.001).

### The proportions and characteristics of admitted patients

In total, 146 of the 680 patients (21.5%) were admitted to in-patient wards, of whom 53 (7.8%) were admitted to in-patient wards at CMHCs and 91 (13.4%) to psychiatric wards in hospitals. There were no significant differences between these two admitted groups in severity of psychiatric and social problems as rated on the HoNOS and GAF. Because the CMHC- and hospital-admitted patients showed no significant differences in severity of psychiatric or social problems, they were included as a single “admitted patient group” for subsequent analysis.

Table 2 shows socio-demographic and clinical characteristics, contacts in the last 48 hours prior to the first consultation with the CRT, and the referral route for patients admitted and not admitted from the CRTs to the in-patient wards. Other than employment status, there were no significant differences in socio-demographic variables. Except for substance abuse, the admitted patients had mental health problems that were significantly more severe on most clinical scales. Those admitted to in-patient wards were also significantly more likely to have been in contact with outpatient clinics during the last 48 hours, and to have been delivered to the CRTs by the police.

**Table 2:** Characteristics and comparison of patients (n = 680) admitted and not admitted to in-patient wards from CRTs

**Patient-level variables**	**Admitted**	**Not admitted**	***p***^**a**^
Overall admission rate to in-patient wards *n* (%)	146 (21.5)	534 (78.5)	
**Socio-demographic variables**			
Age (years): mean (SD)	41.82 (14.66)	39.63 (15.12)	0.124
Gender: n (%) female	84 (58.3)	312 (58.9)	0.908
Single, divorced, or widowed: n (%)	97 (68.3)	318 (60.9)	0.107
Living alone: n (%)	83 (57.6)	313 (58.4)	0.870
Employed at present: n (%)	24 (16.7)	151 (28.2)	0.005
Custody of children aged under 18 years: n (%)	34 (29.1)	134 (34.6)	0.263
**Clinical variables**			
**Clinical diagnosis (ICD–10)**: n (%)			
F10–19 Substance use disorders	8 (5.8)	45 (8.8)	0.260
F20–29 Schizophrenia disorders	28 (20.4)	32 (6.3)	< 0.001
F30–39 Affective disorders	55 (40.1)	165 (32.3)	0.085
F40–49 Neurotic, stress-related and somatoform disorders	22 (16.1)	125 (24.5)	0.037
F60–69 Personality disorders	3 (2.2)	27 (5.3)	0.126
**GAF**: mean (SD)			
Symptom	39.26 (10.31)	50.80 (10.70)	< 0.001
Functioning	41.37 (10.62)	51.78 (12.13)	< 0.001
**Suicide risk**: n (%)			
No suicidal thoughts/death intentions	36 (27.9)	221 (43.0)	0.001
Passive suicidal thoughts, with no death intentions	52 (37.1)	209 (40.7)	0.451
Concrete suicidal plans or self-injury, with no death intentions	43 (30.7)	67 (13.0)	< 0.001
Self-injury with death intentions	6 (4.3)	17 (3.3)	
**Severity of clinical and social problems**: n (%)			
Non-accidental self-injury (HoNOS 2):			
0 No problem	84 (58.3)	410 (76.5)	< 0.001
1 Minor problem requiring no action	18 (12.5)	42 (7.8)	0.080
2 Mild problem but definitely present	13 (9.0)	34 (6.3)	0.260
3 Moderately severe problem	17 (11.8)	31 (5.8)	0.012
4 Severe to very severe problem	12 (8.3)	19 (3.5)	0.014
Substance misuse (HoNOS 3):			
0 No problem	95 (66.0)	388 (72.4)	0.132
1 Minor problem requiring no action	15 (10.4)	52 (9.7)	0.798
2 Mild problem but definitely present	11 (7.6)	39 (7.3)	0.882
3 Moderately severe problem	17 (11.8)	43 (8.0)	0.155
4 Severe to very severe problem	6 (4.2)	14 (2.6)	0.327
Psychotic symptoms (HoNOS 6):			
0 No problem	80 (55.6)	438 (81.7)	< 0.001
1 Minor problem requiring no action	15 (10.4)	48 (9.0)	0.591
2 Mild problem, but definitely present	17 (11.8)	31 (5.8)	0.012
3 Moderately severe problem	21 (14.6)	15 (2.8)	< 0.001
4 Severe to very severe problem	11 (7.6)	4 (0.7)	< 0.001
Depressive symptoms (HoNOS 7):			
0 No problem	16 (11.1)	64 (11.9)	0.784
1 Minor problem requiring no action	21 (14.6)	112 (20.9)	0.090
2 Mild problem, but definitely present	52 (36.1)	219 (40.9)	0.302
3 Moderately severe problem	39 (27.1)	115 (21.5)	0.152
4 Severe to very severe problem	16 (11.1)	26 (4.9)	0.006
**Unwanted incidents**: n (%)			
Attempted suicide	6 (4.2)	8 (1.5)	0.031
Deliberate self-harm	12 (8.3)	20 (3.7)	0.031
Physical attacks on others	5 (3.5)	11 (2.1)	0.351
Physical attacks from others	1 (0.7)	4 (0.7)	
**Past psychiatric history**: n (%)			
Previous mental health service contact	94 (65.2)	307 (57.3)	0.063
Psychiatric admission past 12 months	55 (44.4)	87 (19.0)	< 0.001
**Other characteristics**: n (%)			
Pharmacological treatment	57 (39.6)	184 (34.3)	0.242
Receiving involuntary treatment	1 (0.7)	2 (0.4)	
**Contact/support in 48 hours before admission**: n (%)			
GP	54 (37.5)	176 (32.8)	0.294
Emergency ward	32 (22.2)	101 (18.8)	0.364
Local CMHT	21 (14.6)	62 (11.6)	0.326
Outpatient clinics in CMHC	21 (14.6)	34 (6.3)	0.001
Support from family and/or friends	62 (43.1)	208 (38.8)	0.355
**Referral route to the CRT**: n (%)			
Emergency referrals	114 (79.2)	375 (70.0)	0.029
Self-referrals	31 (21.5)	141 (26.3)	0.242
Delivered to CRT by the police	14 (9.7)	13 (2.4)	< 0.001

^a^*p-*values from chi-squared tests for categorical variables; *t*-tests for continuous variables.

### Team and patient predictors of admission

Table 3 shows the results of the hierarchical logistic regression analysis. According to the bivariate analyses (crude odds ratios), the risk of being admitted to in-patient wards was higher for patients with concrete suicidal plans or self-injury but with no death intention, and for those with mild to very severe psychotic symptoms. The risk was also higher in those who had had a psychiatric admission within the last 12 months or contact or support from a CMHC in the past 48 hours, and for patients delivered to the CRT by the police. In the reduced multivariate model, opening hours were the only significant predictor of admission at the team level. The risk of in-patient admissions was three to five times lower for patients seen by a CRT available outside office hours. The significant patient-level predictors were moderately severe to very severe psychotic symptoms, concrete suicidal plans or self-injury, but with no death intention, and psychiatric admission within the last 12 months. The highest risk of admission was for patients rated by the clinicians as having moderately severe problems (Odds Ratio (OR) = 8.62), or severe to very severe problems (OR = 30.83) with psychotic symptoms. Patients rated with concrete suicidal plans or self-injury but with no death intention by the clinicians had a 6.88 times higher risk of in-patient admission and patients that had been admitted in the past 12 months had a 2.69 times higher risk of in-patient admission. There were no significant interaction effects between the independent variables in the regression model.

**Table 3:** Potential predictors associated with being admitted in logistic regression model (crude and adjusted odds ratios)

**Variables**	**Bivariate model**			**Multivariate model**		
	**Crude OR**^**a**^**for admission**	**95% CI**	***p***^**c**^	**Adjusted OR**^**b**^**for admission**	**95% CI**	***p***^**c**^
**Team level**						
Opening hours (37.5/week = ref)						
Team operates extended hours (70/week)	0.40	0.20–0.79	0.009	0.20	0.08–0.51	0.001
Team operates extended hours (75/week)	0.55	0.36–0.85	0.007	0.36	0.20–0.66	0.001
Team operates extended hours (86/week)	0.58	0.33–1.03	0.063	0.22	0.10–0.49	< 0.001
Number of team members	0.97	0.91–1.03	0.313			
Full-time doctor as part of the team	0.89	0.49–1.61	0.691			
Number of patients included	0.99	0.99–1.00	0.154			
Focus on home treatment	1.39	0.79–2.44	0.256			
Psychosis team in the catchment area	1.13	0.62–2.06	0.685			
Accept self-referral	0.63	0.30–1.33	0.227			
Authority to admit patients to acute in-patient wards	0.80	0.46–1.40	0.437			
**Patient level**						
Age	1.01	1.00–1.02	0.105	1.02	1.00–1.03	0.019
Gender	1.01	0.69–1.47	0.980			
Living alone	0.95	0.64–1.40	0.786			
Employed at present	0.51	0.31–0.82	0.006			
Suicide risk (0 = ref):						
1 Passive death wishes or suicidal thoughts without death intention	1.47	0.92–2.34	0.104	2.29	1.29–4.07	0.005
2 Concrete suicidal plans or self-injury, but without death intention	3.75	2.21–6.33	< 0.001	6.88	2.48–13.63	< 0.001
3 Self-injury with death intention	2.15	0.78–5.88	0.138	3.22	0.78–13.39	0.108
Non-accidental self-injury (HoNOS 2) (0 = ref)						
1 Minor problem requiring no action	2.32	1.25–4.30	0.008			
2 Mild problem, but definitely present	2.09	1.04–4.20	0.040			
3 Moderately severe problem	2.75	1.43–5.28	0.002			
4 Severe to very severe problem	3.40	1.56–7.43	0.002			
Substance misuse (HoNOS 3) (0 = ref)						
1 Minor problem requiring no action	1.27	0.68–2.38	0.459			
2 Mild problem, but definitely present	1.24	0.60–2.54	0.558			
3 Moderately severe problem	1.70	0.92–3.14	0.092			
4 Severe to very severe problem	1.84	0.68–5.00	0.232			
Psychotic symptoms (HoNOS 6) (0 = ref)						
1 Minor problem requiring no action	1.87	0.99–3.55	0.056	3.28	1.54–6.98	0.002
2 Mild problem, but definitely present	3.08	1.60–5.91	0.001	2.33	1.06–5.14	0.037
3 Moderately severe problem	8.37	4.04–17.35	< 0.001	8.62	3.32–22.34	< 0.001
4 Severe to very severe problem	16.46	4.97–54.55	< 0.001	30.83	5.74–165.60	< 0.001
Depressive symptoms (HoNOS 7)						
1 Minor problem requiring no action	0.75	0.36–1.54	0.428			
2 Mild problem, but definitely present	0.94	0.50–1.77	0.847			
3 Moderately severe problem	1.38	0.71–2.69	0.350			
4 Severe to very severe problem	2.77	1.17–6.57	0.021			
Physical attacks on others	1.11	0.58–2.14	0.746			
Psychiatric admission past 12 months	3.85	2.42–6.13	< 0.001	2.69	1.58–4.57	< 0.001
Contact/support from CMHCs in the past 48 hours	3.09	1.63–5.85	0.001	3.78	1.56–9.21	0.004
Self-referral	0.75	0.48–1.19	0.220			
Delivered to CRT by the police	4.00	1.81–8.85	0.001			

^a^crude odds ratios (bivariate analyses); ^b^adjusted odds ratios from a multivariate model reduced by stepwise selection method using Akaike’s information criterion; ^c^ asterisks on *p*-values indicate significant predictors after Holm’s adjustment for family-wise error rates was applied.

## Discussion

We found that about one-fifth of the CRT patients were admitted to in-patient wards, but the CRTs varied in their admission rates. The risk of being admitted was lower for patients seen by CRTs with extended opening hours, and higher for patients with psychotic symptoms, moderate suicidal risk, and a prior history of admission.

Our finding that 21.5% of CRT patients were admitted to in-patient wards confirms previous findings, including those of Brimblecombe et al. (21.1% admitted) and Johnson et al. (22% admitted) [9,14]. This indicates that the Norwegian CRTs achieved one of their goals of providing an alternative to in-patient admission, even though the more severely ill patients were probably under-represented in the sample.

We found no significant differences in the severity of patients’ mental health problems between those admitted to CMHC wards and those admitted to hospital wards. This is somewhat surprising, given that the in-patient units at CMHCs are intended for patients with less severe mental health problems and patients who do not need involuntary admissions. This finding may be related to variations in the capacities of the different in-patient wards, and to patients being admitted wherever beds were available. It may also be related to our previously reported finding that CRTs in Norway probably have a lower proportion of severely ill patients than CRTs in the UK [19]. Patients with less severe illnesses may be more easily admitted to in-patient units in the CMHCs.

The proportions of patients receiving involuntary treatment, delivered to the CRT by the police, or at risk of harming themselves or others, were lower than in similar studies [8,14,15]. This might indicate that a higher proportion of patients with more severe mental illnesses bypass CRTs in their admissions to acute psychiatric wards in Norway than in the UK, although we do not have data to support this assumption. Even though some of these patients could have been treated outside in-patient wards, it must be emphasized that very severely ill patients experiencing imminent risk could not be treated in the community and would warrant direct admission. Some of these situations are very complex to respond to and may be very time-intensive.

### Team and patient predictors of admission

The hierarchical regression analysis results indicate that four CRT team and patient factors were associated with an increased risk for in-patient admission.

Firstly, the lack of extended opening hours was significantly associated with increased risk for in-patient admission. The admission risk was significantly lower for patients seen by CRTs that provided services with extended opening hours on evenings and weekends. In a previous study of the same CRTs, we found that there was a tendency for teams that operated extended opening hours to treat patients with more severe mental illnesses [19]. Patients experience mental health crises in the evenings, at night, and on weekends, and it is difficult for Norwegian CRTs to operate as an adequate alternative to in-patient treatment if they do not operate during these hours. Our findings show that allocation of resources to CRT for extended opening hours increased the proportion of patients for whom CRTs is an alternative to in-patient admission. Apart from CRTs, casualty departments and the acute in-patient wards are the only alternative services for patients experiencing mental health crises outside office hours.

Secondly, the severity of psychotic symptoms was associated with an increased risk of in-patient admission. Being rated as having severe to very severe psychosis on the HoNOS 6 increased the risk of in-patient admission up to 30 times. The symptoms may include poor insight, lack of motivation for treatment, disruptive and threatening behaviour, or other problems that make home treatment impossible.

In the bivariate analysis, delivery to the CRT by the police was significantly associated with in-patient admission, but was not a significant predictor in the adjusted model. This may be because of the relatively small numbers of patients who were delivered to the CRTs by the police (n = 27). The most consistent finding in Cotton et al.’s study was that patients who were uncooperative during their initial assessment were much more likely to be admitted (OR = 10.3) [15]. As with severe psychotic symptoms, presentation with the police is a marker of potential threats of harm to the self or others, and is also potentially related to the level of uncooperativeness, as found by Cotton et al. [15]. Our study might provide some support to their conclusion that uncooperative patients are difficult to treat within the CRT model. For these groups of patients, in-patient treatment will probably continue to be the treatment of choice, despite the implementation of CRTs.

Thirdly, moderate suicidal risk is also associated with increased risk of in-patient admission. The highest level of the suicide scale (self-injury and death intention) was not significant in the model. This potentially surprising finding might be explained by the fact that few patients were rated as having self-injury and death intention (n = 23). Moderate suicidal risk is a marker of potential harm to patients and was often assessed by these Norwegian CRTs as difficult to handle outside the in-patient ward.

Fourthly, psychiatric admission in the past 12 months was associated with increased risk of in-patient admission. Recent psychiatric admission is probably a marker of illness chronicity and persistence, increasing the likelihood of new crisis episodes. Previous in-patient treatment may also have established expectations among patients and caregivers about in-patient treatment; in-patient care may be seen as necessary to manage acute episodes, resulting in patients and caregivers expecting admission rather than care delivered in the community by the CRT.

There is a gap in the literature regarding the effect of specific variations in the staffing and practices of CRTs on admission rate [5]. Cotton et al. found the particular CRT delivering the service was a consistent determinant for hospital admission [15], but this variable was not otherwise specified in their study. Even though there are no fidelity criteria for the organization and operations of the CRT model, we included several team-level variables considered important for effective CRT care in the literature [1,33]. Except for opening hours, the hierarchical logistic regression analysis did not show any significant team-level predictors associated with admission to in-patient wards. This was surprising as there were significant variations between the CRTs in several aspects, such as admission practice, staffing, and focus on home treatment. This may suggest that similar patients experiencing similar levels of acuity may be treated in a similar way across different CRTs in Norway.

There may be a difference between the UK and Norway with respect to the admission threshold of CRTs. Contrary to the findings of Cotton et al. [15], in our study, clinical variables, such as psychotic symptoms, suicidal risk, and previous admissions, increased the likelihood of in-patient admission. Our findings are similar to those of earlier studies of home treatment as an alternative to hospital admission [14,16,17] conducted before the National Health Service Plan in the UK [3]. Cotton et al.’s findings of clinical variables as non-predictors may indicate that the CRTs in the UK, to a larger extent, provide help for more severely ill patients without in-patient admission. Differences in the gate-keeping function for acute beds and 24/7 availability of CRTs are probably important factors that contribute to the treatment of more severely ill patients without in-patient admission. However, recent psychiatric admission and experience of severe psychotic symptoms are often markers of severe mental health problems, and patients with these symptoms may be more difficult to treat in the community. For such patients, psychiatric admission may be the most suitable option for care. Perhaps the Norwegian CRTs ensure that those patients most likely to require containment and intensive treatment are admitted to an in-patient ward. On the other hand, the CRTs in the UK may be more experienced because the model is more established there. It is therefore possible that they manage patients with more severe mental health problems outside in-patient wards in a satisfactory manner.

A further factor that may potentially explain the admission rate is the extent to which teams permit patients to choose whether to go to hospital. Current formulations of the model suggest that home treatment should be delivered whenever feasible, but the increasing emphasis on allowing service users a choice conflicts with these imperatives [15].

A range of other contextual factors not included in this study may also determine the types of patients admitted to a particular unit at a given time. These include the availability of affordable housing, prioritization at emergency departments, and the availability of mental health services in the community [34].

### Strengths and limitations

The main strength of our study is that the inclusion of all patients presenting at a CRT during the study period provides good external validity. In addition, a hierarchical logistic regression model took into account both patient- and team-level variables in assessing the risk of admission to in-patient wards from CRTs. Causality cannot be determined because this was not a randomized controlled trial. The 62 clinical raters in this study may have contributed to unpredictable error variance in the data material. The data for different teams were collected at different points in time and there may be seasonal variation in admissions or other exogenous factors that were not controlled for in our study.

## Conclusion

The CRTs achieved one of their goals of providing an alternative to in-patient admission for about four-fifths of their patients, but the CRTs varied in their admission rates. The risk of being admitted to an in-patient ward was significantly lower for patients seen by CRTs with extended opening hours, which illustrates the importance of CRTs offering an extended-hours service. Patients with psychotic symptoms, moderate suicidal risk, and a prior history of admission had a higher risk of in-patient admission. Even though some severely ill patients need in-patient care, the CRTs may give care in the community for suicidal and psychotic patients, as well as for patients with relapsing mental illnesses.

## Competing interests

The authors declare that they have no competing interests.

## Authors’ contributions

TR, RWG, and NH designed the study and formulated the research questions. NH conducted the literature search. NH and JS-B performed the statistical analysis and interpreted the data, with significant support from SJ and TR. The manuscript was written by NH and substantially revised by TR and SJ. The final version of the manuscript was prepared and revised by all authors. TR was the head supervisor of the manuscript and the project leader of the Multicentre Study of Acute Psychiatry in Norway (MAP). All authors have read and approved the final manuscript.

## Pre-publication history

The pre-publication history for this paper can be accessed here:

http://www.biomedcentral.com/1471-244X/13/117/prepub
